# Gammaherpesvirus in Cervid Species from Norway: Characterization of a New Virus in Wild and Semi-Domesticated Eurasian Tundra Reindeer (*Rangifer tarandus tarandus*)

**DOI:** 10.3390/v12080876

**Published:** 2020-08-11

**Authors:** Carlos G. das Neves, Carlos Sacristán, Knut Madslien, Morten Tryland

**Affiliations:** 1 Norwegian Veterinary Institute, P.O. Box 750 Sentrum, NO-0106 Oslo, Norway; carlosvet.sac@gmail.com (C.S.); knut.madslien@vetinst.no (K.M.); 2 Department of Arctic and Marine Biology, UiT The Arctic University of Norway, N-9019 Tromsø, Norway; morten.tryland@uit.no

**Keywords:** Fennoscandia, gammaherpesvirus, moose, red deer, reindeer, ungulates, virology, wildlife diseases, virus discovery

## Abstract

Gammaherpesvirus infections have been described in cervids worldwide, mainly the genera *Macavirus* or *Rhadinovirus*. However, little is known about the gammaherpesviruses species infecting cervids in Norway and Fennoscandia. Blood samples from semi-domesticated (*n* = 39) and wild (*n* = 35) Eurasian tundra reindeer (*Rangifer tarandus tarandus*), moose (*Alces alces*, *n* = 51), and red deer (*Cervus elaphus*, *n* = 41) were tested using a panherpesvirus DNA polymerase (DPOL) PCR. DPOL-PCR-positive samples were subsequently tested for the presence of glycoprotein B (gB) gene. The viral DPOL gene was amplified in 28.2% (11/39) of the semi-domesticated reindeer and in 48.6% (17/35) of the wild reindeer. All moose and red deer tested negative. Additionally, gB gene was amplified in 4 of 11 semi-domesticated and 15 of 17 wild Eurasian reindeer DPOL-PCR-positive samples. All the obtained DPOL and gB sequences were highly similar among them, and corresponded to a novel gammaherpesvirus species, tentatively named *Rangiferine gammaherpesvirus 1*, that seemed to belong to a genus different from *Macavirus* and *Rhadinovirus.* This is the first report of a likely host-specific gammaherpesvirus in semi-domesticated reindeer, an economic and cultural important animal, and in wild tundra reindeer, the lastpopulation in Europe. Future studies are required to clarify the potential impact of this gammaherpesvirus on reindeer health.

## 1. Introduction

Herpesviruses are large DNA viruses which are able to establish latency in infected hosts. Three different subfamilies of herpesvirus are recognized within the family *Herpesviridae* as follows: *Alphaherpesvirinae*, *Betaherpesvirinae,* and *Gammaherpesvirinae* (ICTV, 2020) [1]. Different species in the subfamilies *Alpha-*, *beta-,* and *Gammaherpesvirinae* infect cervids [2,3]. Among alphaherpesviruses, cervid alphaherpesvirus 1 (CvHV1) and cervid alphaherpesvirus 2 (CvHV2) are well documented in different cervid species and populations, with CvHV2 being associated with infectious keratoconjunctivitis and possibly with respiratory diseases in semi-domesticated Eurasian tundra reindeer (*Rangifer tarandus tarandus*) [4,5,6]. Regarding betaherpesviruses, a novel species, designated cervid herpesvirus 3 was detected in eye swabs of reindeer with ocular lesions [2].

The subfamily *Gammaherpesvirinae* is divided into the following four genera: *Lymphocryptovirus*, *Rhadinovirus*, *Percavirus,* and *Macavirus*. The genus *Macavirus* contains nine recognized virus species [7], while several other species have also been reported [8,9]. Most viruses in this genus are closely related both genetically and antigenically and are associated with the disease malignant catarrhal fever (MCF) in different ruminant species. Several gammaherpesviruses have been reported in cervids, some of them responsible for MCF, for example, ovine gammaherpesvirus 2, caprine gammaherpesvirus 2, the malignant catarrhal fever virus (MCFV) of white-tailed deer (*Odocoileus virginianus*), and the alcelaphine herpesvirus 2-like virus; the latter responsible for MCF in Barbary red deer (*Cervus elaphus barbarus*) [1,8,10,11,12]. Many other gammaherpesviruses have been reported in wild ruminants, but due to limited information were named ruminant rhadinovirus, depicting an older nomenclature of ruminant rhadinovirus type 1 (Type 1 RuRv-MCF like) and type 2 (Type 2 RuRv-lymphotropic like [13]), based on shared antigenic epitopes.

MCF is one of the most concerning diseases caused by gammaherpesvirus, affecting domestic and wild ruminants of the families Bovidae and Cervidae [11,14], and pigs (*Sus scrofa domestica*) [15]. Despite being able to cause severe disease in non-adapted species [3], MCFV infection is usually of low pathogenicity in specific natural hosts, as described for ovine gammaherpesvirus 2 in domestic sheep (*Ovis aries*), and caprine gammaherpesvirus 2, and possibly white-tailed deer MCFV in goats (*Capra aegagrus hircus*) [12,16,17,18].

Several species of cervids inhabit Fennoscandia, i.e., Eurasian tundra reindeer, moose (*Alces alces*), red deer (*C. elaphus atlanticus*), fallow deer (*Dama dama*), roe deer (*Capreolus capreolus*), and white tailed deer (*Odocoileus virginianus*, introduced in Finland) [19,20,21]. Eurasian reindeer live as wild animals in 23 fragmented subpopulations in mountain areas of southern Norway, whereas semi-domestic reindeer are mainly found in the northern part of the country [22,23]. Moose inhabit the whole country, but with lower population densities on the west coast and in northern Norway [23]. Red deer inhabit the southern part of Norway and are especially prominent along the west coast, while roe deer are found in almost all areas of the country, but less frequently in the north [23]. Fallow deer comprises a restricted population in the southeastern part of the country. The distribution of some of these cervid species overlaps with the habitat used by domestic species, mainly sheep, promoting shared grazing areas [24].

In Norway, MCF cases have been reported in cattle (*Bos taurus*) and pigs [15,25], and fatal outcomes associated with ovine gammaherpesvirus 2 and caprine gammaherpesvirus 2 have also occurred in moose, roe deer, and red deer [11]. A serosurvey of sheep and goats in Norway indicated a MCFV seroprevalence of almost 100% in both species [25]. A few cases of fatal MCF have been reported in captive reindeer from the United States [26], and, recently, also in a semi-domesticated reindeer in northern Norway [27]. Additionally, a serological study using a competitive ELISA (cELISA) to detect specific antibodies against MCFV in a group on 3339 apparently healthy semi-domesticated reindeer from Finnmark County, Norway [28], indicated an overall seroprevalence of 3.5%.

In spite of previous reports available in the literature, information regarding the gammaherpesvirus species infecting wild cervids from Fennoscandia are limited. In light of the potential spillover of these viruses from domestic animals to wild populations and the serological evidence of one or more circulating gammaherpesviruses in Norway, our aim was to surveying for the presence of gammaherpesviruses DNA in blood samples of wild Eurasian reindeer, semi-domesticated reindeer, moose, and red deer from Norway, attempting to characterize potentially novel herpesviruses.

## 2. Materials and Methods

### 2.1. Samples

**Figure 1:**
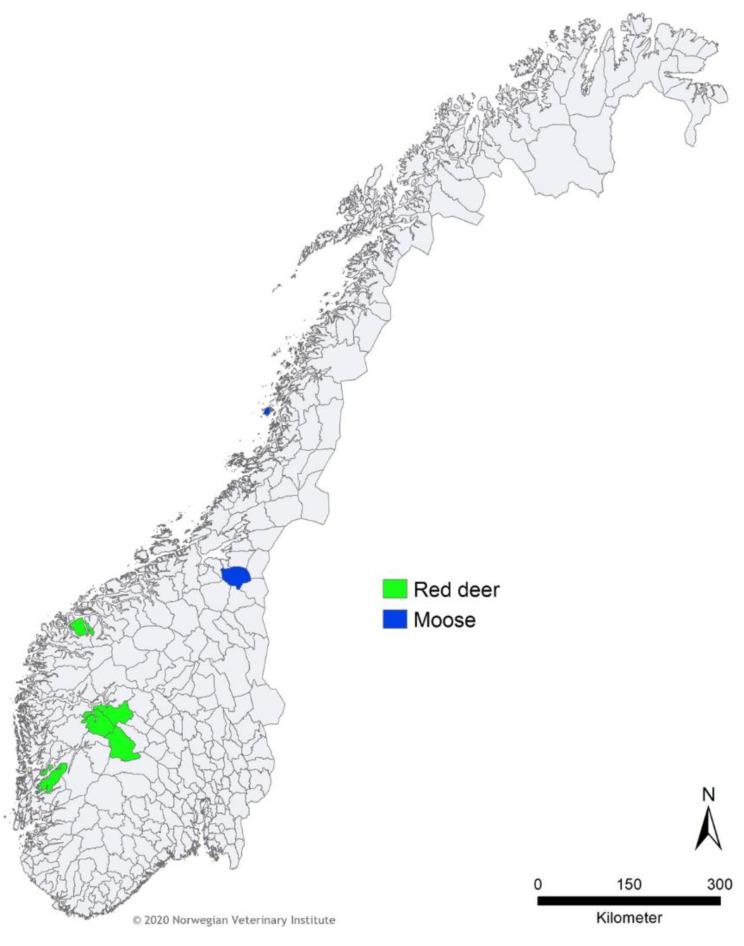
Location of the moose (*Alces alces*) and red deer (*Cervus elaphus*) samples tested in this study and which were all PCR negative. The map depicts Norway’s municipalities.

**Figure 2:**
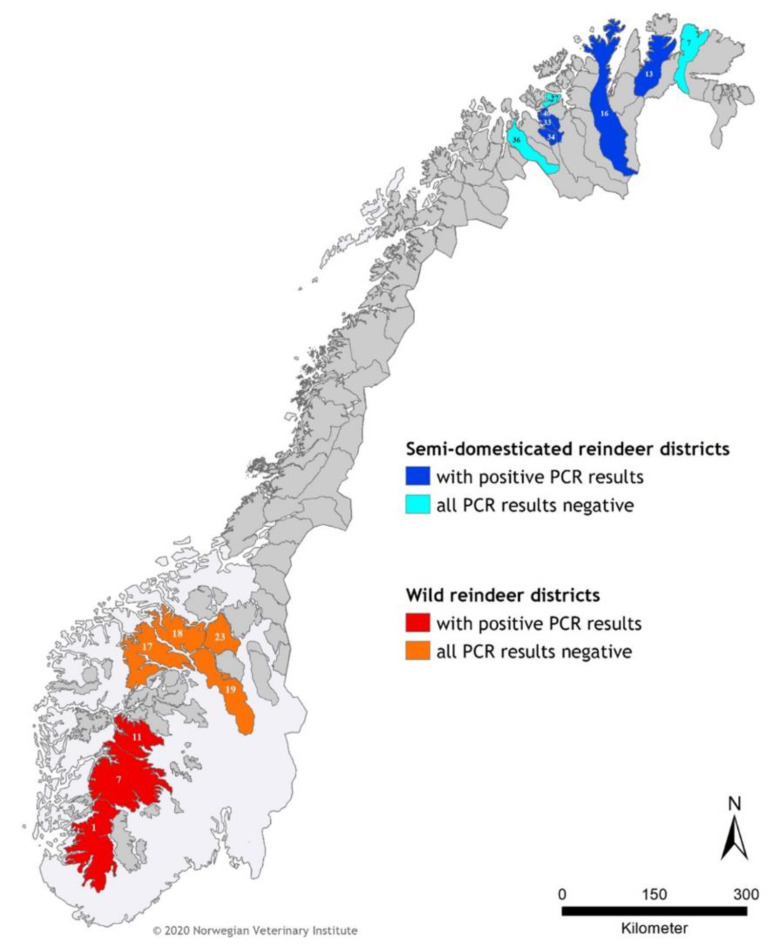
Location of the wild and semi-domesticated Eurasian tundra reindeer (*Rangifer tarandus tarandus*) samples tested in this study. The map depicts in red/orange wild reindeer management areas, and in dark/light blue semi-domesticated reindeer husbandry districts. Numbers in the areas/districts refer to information from Table 1.

Blood samples from thirty-nine (*n* = 39) semi-domesticated reindeer from eight reindeer herding districts in Finnmark County, northern Norway, were selected based on a previous serological survey, on samples obtained between 2004 and 2006, and in 2009 [28]. These included samples tested seropositive (*n* = 23) and negative (*n* = 16) for gammaherpesvirus by a direct cELISA. Additionally, blood samples were collected from wild reindeer (*n* = 35), red deer (*n* = 41), and moose (*n* = 51), chemically restrained or hunted between 2014 and 2018 in different Norwegian regions (Hardangervidda, Lesja, Nordfjella, Oppdal, Rondane sør, Setesdal Ryfylke, and Sunndal for reindeer; Aurland, Hol, Kvinnherad, Lærdal, and Ørstad for red deer; and Selbu and Vega for moose). Some of the reindeer samples were collected as part of a national project to cull the wild reindeer population of Nordfjella, the site of the first reported case of chronic wasting disease in Europe [29]. No information on the gammaherpesvirus serological status was available for wild reindeer, red deer, and moose. A summary of all samples, species, and locations is provided in Table 1 and Figure 1 and Figure 2.

### 2.2. Molecular Diagnostics

In order to lyse the blood samples, 200 µL of whole blood samples preserved in EDTA were macerated with 350 of ATL lysis buffer (Qiagen, Hilden, Germany) and a tungsten carbide bead (Qiagen) for 10 min. Subsequently, total DNA was extracted using the DNeasy Blood and Tissue kit (Qiagen) (semi-domestic reindeer) or the Qiasymphony DSP virus/pathogens midi kit (Qiagen) (wild reindeer, moose, and red deer) in the QIAsymphony automated extraction system (Qiagen), according to the manufacturer’s instructions. We employed the panPCR designed by VanDevanter and colleagues to amplify a fragment of 250 bp of herpesviral DNA polymerase (DPOL) gene [30]. The primer set GH1 was used to amplify a fragment of approximately 500 bp of glycoprotein B (gB) gene on DPOL-positive samples [31]. Controls (ovine gammaherpesvirus 2 and no template control, respectively) were included in each PCR assay. Following 2.0% agarose gel electrophoresis, several PCR products within the expected size range were purified with ExoSAP-IT Express (USB Corporation, Cleveland, OH, USA) and directly sequenced in both directions.

Visual inspection determined the chromatogram sequence quality. The obtained sequences were aligned using ClustalW 2 on Mega 7.0 [32] and were compared to similar ones from GenBank using the Blast search tool. In order to establish the percentage of nucleotide and amino acid identity between the obtained and reference sequences, p-distance analyses were performed on Mega 7.0. [32].

**Figure 3:**
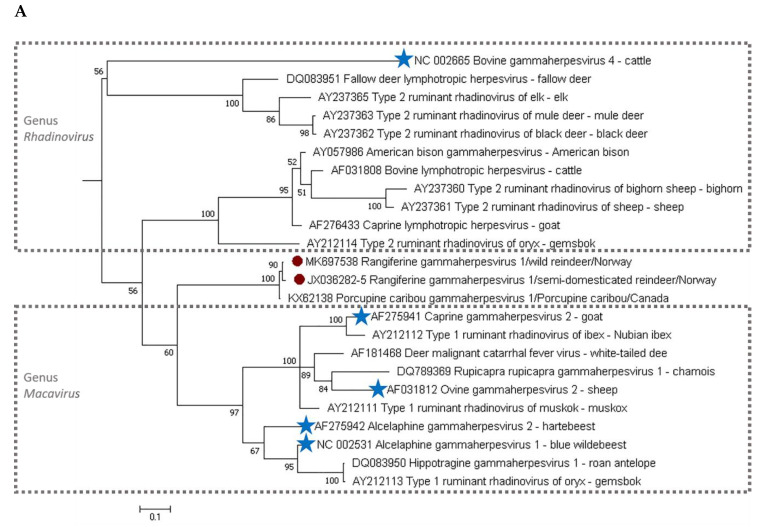

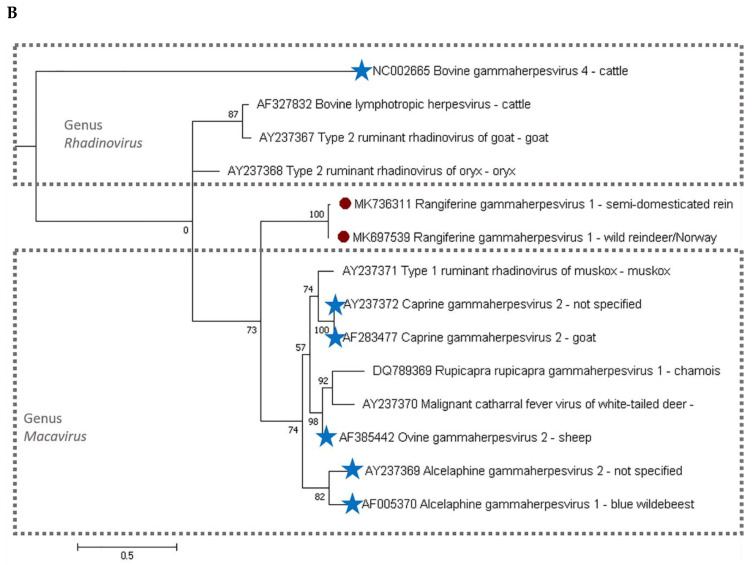
MrBayes Bayesian inference phylogenetic tree of a ClustalW alignment of gammaherpesvirus DNA polymerase nucleotide sequences (**A**) and gammaherpesvirus DNA glycoprotein B sequences (**B**). ● represents the reindeer sequences obtained in this paper. Dotted grey boxes represent the two gammaherpesvirus genera depicted in this analysis. One should note that the genus *Macavirus* has been established as a new denomination for a previous type 1 rhadinovirus (malignant catarrhal fever (MCF)-like) group. This explains the presence of some rhadinovirus names in sequences now included in the *Macavirus* genus. ★ represents sequences of viral species officially classified in its respective genus by the International Commission for Taxonomy of Viruses (ICTV), whereas all other sequences are classified tentatively (from GenBank taxonomy lists). The scale bar indicates the number of nucleotide substitutions per site.

**Figure A1:**
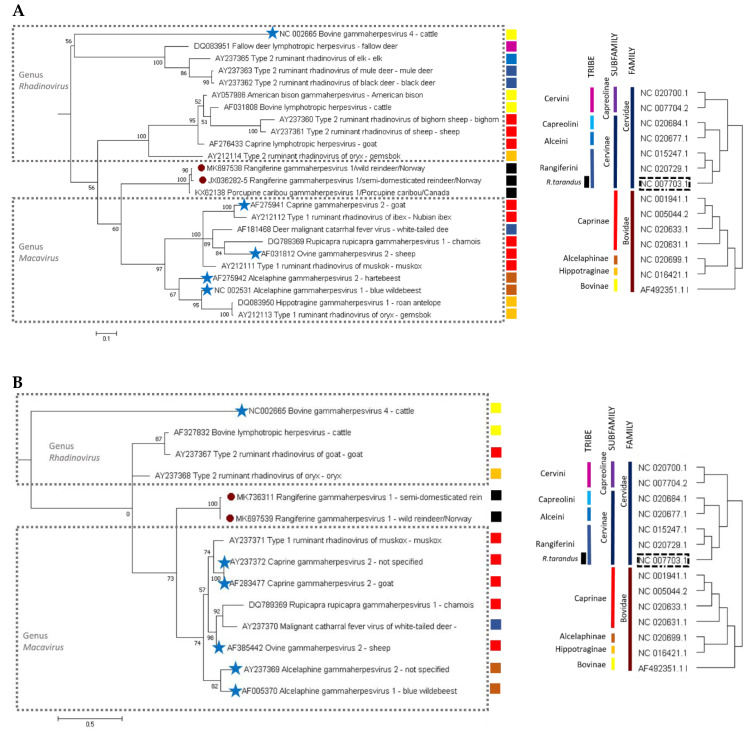
The information on the virus phylogenetic trees (**A**,**B**) is already presented in Figure 3. In front of each viral sequence, a colored rectangle refers to the taxonomic classification of its host species. These colors are explained on the right-hand side of the figure in an evolutionary cladogram based on species within the Cervidae and Bovidae families. Colored bars indicate the families, subfamilies, and tribes relevant to host species of viral sequences analyzed in this paper. The sequence referring to reindeer is highlighted in a black dashed box. The evolutionary relationships of taxa were inferred using the minimum evolution method [48]. The optimal tree with the sum of branch length = 0.91536236 is shown. The evolutionary distances were computed using the maximum composite likelihood method [49] and are in the units of the number of base substitutions per site. The ME tree was searched using the close-neighbor-interchange (CNI) algorithm at a search level of 1. The analysis involved 14 complete mitochondrion genome sequences (average of 16,000 bp). All positions containing gaps and missing data were eliminated. There was a total of 11,199 positions in the final dataset. Evolutionary analyses were conducted in MEGA7 [32]. For information on the ascension numbers used on the taxa cladogram, please refer to Table A1.

The phylogenetic analyses were performed based on the DPOL and gB gene nucleotide sequences obtained in this study and available corresponding sequences of other gammaherpesvirus from wild and domestic ruminants (Figure 3). Bovine gammaherpesvirus 4 sequences were selected as outgroups to help generate Bayesian inference phylogenetic trees. Initially, multiple sequence alignments were performed using the ClustalW algorithm on Mega 7.0 [32]. In order to select the best model of evolution, the jModelTest2 program (version 2.1.10, [33]) was used prior to the construction of the Bayesian phylogenetic trees for DPOL and gB genes, under the Akaike Information Criterion (AIC). The Bayesian analyses were carried out on the platform Phylogeny.fr (http://www.phylogeny.fr/ [34]) using MrBayes program v3.2.6, [35]. We ran Markov Chain Monte Carlo (MCMC) chains for 10,000 generations, sampling every 10 generations. A burn-in of 1000 was applied. Finally, a 50% majority rule consensus tree was constructed with branching support shown as posterior probability percentages. An extended version of Figure 3 with information on the taxonomic classification of the host species from which viral sequences were obtained and is presented in Appendix A in Figure A1 and Table A1.

## 3. Results

The viral DPOL gene was amplified from 11 out of 39 (28.2%) semi-domesticated reindeer samples, and four of the amplified products were submitted for sequencing. The gB gene was amplified in four out of the 11 DPOL positive animals, and gB amplicons were also submitted for sequencing.

Viral DNA amplification of the DPOL gene was obtained in 17 of 35 (48.6%) wild reindeer blood samples, of which 12 were sequenced. All positive animals belonged to subpopulations from the adjacent territories of Hardangervidda, Setesdal Ryfylke, and Nordfjella. Furthermore, the gB gene PCR amplified viral DNA in 15 of these 17 samples, all of them generating high quality sequences. The novel DPOL and gB viral unique sequences were deposited in GenBank, for semi-domesticated reindeer (DPOL, JX036282 to JX036285 and gB, MK736311) and for wild reindeer (accession number MK697538 and MK697539, respectively).

There was no viral DNA amplified from any of the moose or red deer samples. All results are summarized in Table 1 and Figure 1, Figure 2 and Figure 3. For semi-domesticated reindeer, Table 2 summarizes the relation between positive and negative PCR results and the results obtained in a previous serological survey.

A nucleotide identity of 99% was observed between our wild and semi-domesticated reindeer DPOL sequences (approximately 250 bp), corresponding to an amino acid identity of 100%, whereas 100% nucleotide identity amongst them was observed for gB (approximately 500 bp). The obtained sequences were clearly different from other ruminant gammaherpesvirus sequences available from GenBank (Figure 3). The highest DPOL nucleotide (99.4%) and deduced amino acid (98.2%) similarity was to a sequence of gammaherpesvirus identified in a porcupine caribou (*Rangifer tarandus granti*) from Yukon, northwestern Canada (KX062138, ANC35073). This was followed by another gammaherpesvirus from porcupine caribou (KX062139, ANC35074) of the same region (nucleotide and amino acid identities of 96% and 93%, respectively).

The gB sequences from reindeer presented the highest nucleotide (74.7%) and amino acid (82.4%) identities, respectively, a gammaherpesvirus obtained from muskox (*Ovibos moschatus*, AY237371) and an alcelaphine gammaherpesvirus 2 sequence from a topi (*Damaliscus lunatus jimela*, YP_009044395.1). Both DPOL and gB sequences indicate a potential novel gammaherpesvirus in reindeer.

## 4. Discussion

On the basis of this study’s detection of gammaherpesvirus DNA in wild and semi-domesticated reindeer and its phylogenetic differences as compared with other gammaherpesvirus sequences, one can consider that a novel gammaherpesvirus species, tentatively named *Rangiferine gammaherpesvirus 1*, is circulating within the reindeer populations in Norway. The proposed name refers to the cervid genus, therefore, more accurate than reindeer gammaherpesvirus 1 or porcupine reindeer gammaherpesvirus and follows the recommendations of the International Committee on Taxonomy of Viruses [7]. The tentative “Rangiferine gammaherpesvirus” does not, however, seem to be properly grouped either within the MCF type of viruses (*Macavirus* genus) or within several rhadinoviruses of wild ruminants (*Rhadinovirus* genus), as shown in both phylogenetic trees (Figure 3). To the authors’ knowledge, this is the first report of what seems to be a host-specific gammaherpesvirus in Eurasian tundra reindeer (*R. tarandus tarandus*). Another gammaherpesvirus, ovine herpesvirus 2, has been previously amplified from the semi-domesticated reindeer with clinical MCF [27]. The sequences in semi-domesticated reindeer reported in our study, while only now published, predate the only other report of gammaherpesviruses in porcupine caribou from Canada [36]. Nevertheless, the phylogenetic analyses confirm that the gammaherpesvirus sequences found in Canada are highly similar to the ones circulating in Norway (based only on DPOL, since no information on gB is available from Canada). This further strengthens the theory that reindeer are probably the natural host for this novel gammaherpesvirus species, and is in line with a serological survey conducted in northern Norway that found a low (3.5%, *n* = 3339) yet geographically widely spread prevalence of gammaherpesvirus in semi-domesticated reindeer [28]. A similar seroprevalence for gammaherpesvirus has also been found in a recent study in Norway that screened reindeer herds spreading from the southern to northern regions of the country [37]. Furthermore, the absence of apparent clinical disease, in all animals from where this virus was amplified, reinforces the theory of reindeer as the natural host, since the pathogenicity of gammaherpesviruses can be very often limited in their natural host [38]. Nevertheless, future studies are necessary in order to verify this hypothesis.

The presence of an endemic gammaherpesvirus in reindeer is relevant for both wild and semi-domesticated populations. Future studies should address if serological cross-reactions between this novel virus and other gammaherpesviruses, in serological tests designed to detect if malignant catarrhal fever exist, could result in false positive results that could trigger containment measures with potential economic consequences. Previous studies used a competitive ELISA with an antigen based on a conserved epitope among all known members of the malignant catarrhal fever virus group. These studies showed 5.9% seropositivity in captive Eurasian reindeer from Germany (*n* = 119) [39], 4% in wild Eurasian reindeer from Norway (*n* = 250) and porcupine caribou from Alaska (*n* = 232) [40], and 3.5% (*n* = 3339) in apparently healthy, semi-domesticated Eurasian reindeer from Finnmark County, northern Norway [28]. Such findings contrasted with the prevalence we obtained in this study by PCR (28.2% and 48.6% in semi-domesticated and wild reindeer, respectively). This was similar to PCR prevalence results observed in whole blood samples from several other ruminant species, for example, 42.9% (six of 14) in Dall sheep (*Ovis dalli*) [40], and 72% (23/32) in peripheral blood mononuclear cells of clinically healthy captive white-tailed deer (*Odocoileus virginianus*) [41]. The possibility that the antigens in the available serological test did not sufficiently cross react with antibodies against *Rangiferine gammaherpesvirus 1* cannot of course be discarded. Similarly, none of the black-tailed deer (*Odocoileus hemionus*), mule deer (*Odocoileus hemionus*), elk (*C. elaphus canadensis*), or addax (*Addax nasomaculatus*) serum samples from infected animals with novel gammaherpesvirus species, and different from those of the malignant catarrhal fever virus group, were antibody positive when tested by cELISA [13].

As shown in Table 2, crossing serology and PCR results allow us to draw some additional interesting inferences as follows: Of the 14 PCR-positive semi-domesticated reindeer, 11 were seropositive, possibly indicating reactivation of a latent infection, because in general, when an immune response is detected the viremia stage has already subsided. Latency is a known characteristic of herpesviruses associated with lifelong infections and immune evasion, and usually is the result of a long co-evolution with their natural host, as it requires the virus to circularize and form an episomal DNA element packed in histones and copied by cellular DNA polymerases, along with the chromosomes [42]. If these PCR and serology positive animals truly are cases of viral reactivation, this could be additional evidence of this virus being a reindeer specific gammaherpesvirus that has co-evolved with the host over a long period. The remaining three PCR positive, serology negative animals could most likely be the result of technical limitations of the serological assay. Considering that both DPOL and gB PCRs target a highly conserved region of the viral genome, and that both produce relatively short amplicons, one can hypothesize that the lower number of PCR positives for gB in relation to DPOL is more likely to be the result of a lower efficiency of gB PCR method rather than mismatches in the primer regions. Since both PCR methods are commonly used for the detection and characterization of new gammaherpesviruses, a re-evaluation of the gB method and its efficiency warrants some attention.

All moose and red deer samples tested in this study were herpesvirus-PCR-negative. In a previous study, ovine herpesvirus 2 was detected in brain, spleen, or lymph node samples from three moose with lethal MCF, while spleen samples from clinically healthy moose (*n* = 23) and red deer (*n* = 17), all hunted on the Norwegian region of Lesja, were negative to the same panherpesvirus PCR used here [43]. Overall, in Norway, ovine gammaherpesvirus 2 and caprine gammaherpesvirus 2 have been detected, respectively, in eight moose and one red deer, and in two moose with MCF lesions [11,43]. Additionally, lesions consistent with MCF were reported in two moose from Sweden [44], and another two moose from Canada presented ovine gammaherpesvirus 2-associated MCF [45]. Furthermore, specific gammaherpesviruses have been detected in deer, including captive red deer from New Zealand [46], elk (unidentified subspecies) from the United States [13], and elk (*C. canadiensis*, previously named *C. elaphus canadensis*) from Canada [36,47]. Additionally, Barbary red deer (*C. elaphus barbarus*) infected with a gammaherpesvirus closely related to alcelaphine gammaherpesviruses 2 reportedly developed clinical signs similar to those of MCF [10]. The absence of viral DNA amplification in our red deer and moose cases could, among others, be due to a true absence of the virus or a low prevalence within the populations. The more solitary behavior of moose and deer in contrast to reindeer can also imply fewer contacts and transmission events. Additionally, the fact that the proposed *Rangiferine gammaherpesvirus 1*, identified in reindeer across Norway, was absent in other cervid species, despite the populations’ overlap in some areas, could indicate that moose and red deer are either not susceptible to this new virus infection or that latency is not successful in these species. The absence of latency could well hinder the detection by PCR in apparently healthy animals, as observed in those included in this study. We recognize, however, that we have a geographic limited sampling, especially for moose (only two municipalities), and analyzing more animals from different locations in the country would be necessary to draw conclusions on species susceptibility to this new virus.

Herein, we described infection by a novel gammaherpesvirus, tentatively named *Rangiferine gammaherpesvirus 1*, in an economic and cultural important species (semi-domesticated reindeer) and in the last wild Eurasian tundra reindeer population in Europe. Our findings contribute to the knowledge of infectious agents affecting this taxon. Future studies are warranted to elucidate on cross-reactions with current MCF serological tests after infection with *Rangiferine gammaherpesvirus 1*, as well as the pathological potential and impact of this novel virus on reindeer populations and possibly other cervid species and domestic ruminants.

## Figures and Tables

**Table 1:** Results for the gammaherpesvirus PCRs conducted in this study presented by species, and district/municipality of origin (wild cervids) or reindeer-herding district in Finnmark County (semi-domestic reindeer).

Species	Municipality (for Red Deer and Moose) or Reindeer District	Number of Samples	Gammaherpesvirus PCR	
			DPOL +	gB +
**Semi-domesticated reindeer**	7 ZE Rákkonjárga	3	0	0
	13ZG Lágesduottar/Ifjordfjellet	3	1	0
	16 ZS Kárašjoga oarjjabealli Karasjok vestre	10	4	2
	27 YK Joahkonjárga	3	0	0
	33YP Spalca	1	1	1
	34 YR Ábborašša	3	1	0
	36 YT Cohkolat ja Biertavárri	1	0	0
	40 YX Orda	15	7	1
	subtotal	39	14	4
**Wild reindeer**	1 Setesdal Ryfylke	4	2	1
	7 Hardangervidda	13	9	8
	11 Nordfjella	10	6	6
	17 Reinheimen-Breheimen	1	0	0
	18 Snøhetta	1	0	0
	19 Rondane sør	2	0	0
	23 Knutshø	4	0	0
	subtotal	35	17	15
**Red deer**	Aurland	4	0	0
	Hol	6	0	0
	Kvinnherad	11	0	0
	Lærdal	16	0	0
	Ørstad	4	0	0
	subtotal	41	0	0
**Moose**	Selbu	24	0	0
	Vega	27	0	0
	subtotal	51	0	0
	**Total**	**166**	**31**	**19**

**Table 2:** Relation between PCR and serology results for the semi-domesticated Eurasian tundra reindeer (*Rangifer tarandus tarandus*) samples included in this study. The serology results refer to a previous survey published in 2013, where 116 out of 3339 (3.5%) apparently healthy semi-domesticated reindeer scored positive for specific antibodies to the malignant catarrhal fever virus (MCFV) group by a direct competitive inhibition enzyme-linked immunosorbent assay [28].

		cELISA		
		POS	NEG	TOTALS
**PCR Results**	DPOL +/gB +	4	0	4
	DPOL +/gB −	7	3	10
	DPOL −/gB +	0	0	0
	DPOL −/gB −	12	13	25
	TOTALS	23	16	39

## Appendix A

Figure A1 (A and B) as well as Table A1 provide additional information on the taxonomy of the host species from which the viral sequences used in this paper were obtained.

**Table A1:** Information on the different species within the Cervidae and Bovidae families used on this analysis and retrieved from GenBank at the National Center for Biotechnology information.

GenBank Accession Number	Organism	Common Designation
NC_020700.1	*Dama dama*	Fallow deer
NC_007704.2	*Cervus elaphus*	Red deer
NC_020684.1	*Capreolus capreolus*	Roe deer
NC_020677.1	*Alces alces*	Moose/elk
NC_015247.1	*Odocoileus virginianus*	White-tailed deer
NC_020729.1	*Odocoileus hemionus*	Mule deer
NC_007703.1	*Rangifer tarandus*	Reindeer
NC_001941.1	*Ovis aries*	Sheep
NC_005044.2	*Capra hircus*	Goat
NC_020633.1	*Rupicapra rupicapra*	Chamois
NC_020631.1	*Ovibus moschatus*	Muskox
NC_020699.1.	*Connochaetes taurinus*	Blue wildebeest
NC_016421.1	*Oryx dammah*	Scimitar oryx
AF492351.1	*Bos Taurus*	Bovine/Cattle
